# Fault Diagnosis Strategies for SOFC-Based Power Generation Plants

**DOI:** 10.3390/s16081336

**Published:** 2016-08-22

**Authors:** Paola Costamagna, Andrea De Giorgi, Alberto Gotelli, Loredana Magistri, Gabriele Moser, Emanuele Sciaccaluga, Andrea Trucco

**Affiliations:** 1 Department of Civil, Chemical and Environmental Engineering (DICCA), University of Genoa, Genova 16145, Italy; paola.costamagna@unige.it; 2 Department of Electrical, Electronic, Telecommunications Engineering, and Naval Architecture (DITEN), University of Genoa, Genova 16145, Italy; andrea.degiorgi@edu.unige.it (A.D.G.); alberto.gotelli@gmail.com (A.G.); gabriele.moser@unige.it (G.M.); emanuelesciaccaluga@libero.it (E.S.); 3 Department of Mechanics, Energetics, Management, and Transportation (DIME), University of Genoa, Genova 16145, Italy; loredana.magistri@unige.it; 4 Pattern Analysis & Computer Vision (PAVIS), Istituto Italiano di Tecnologia (IIT), Genova 16163, Italy

**Keywords:** solid oxide fuel cell (SOFC), quantitative modelling, fault detection and isolation (FDI), model-based and data-driven strategies, pattern recognition, random forest (RF)

## Abstract

The success of distributed power generation by plants based on solid oxide fuel cells (SOFCs) is hindered by reliability problems that can be mitigated through an effective fault detection and isolation (FDI) system. However, the numerous operating conditions under which such plants can operate and the random size of the possible faults make identifying damaged plant components starting from the physical variables measured in the plant very difficult. In this context, we assess two classical FDI strategies (model-based with fault signature matrix and data-driven with statistical classification) and the combination of them. For this assessment, a quantitative model of the SOFC-based plant, which is able to simulate regular and faulty conditions, is used. Moreover, a hybrid approach based on the random forest (RF) classification method is introduced to address the discrimination of regular and faulty situations due to its practical advantages. Working with a common dataset, the FDI performances obtained using the aforementioned strategies, with different sets of monitored variables, are observed and compared. We conclude that the hybrid FDI strategy, realized by combining a model-based scheme with a statistical classifier, outperforms the other strategies. In addition, the inclusion of two physical variables that should be measured inside the SOFCs can significantly improve the FDI performance, despite the actual difficulty in performing such measurements.

## 1. Introduction

Fuel cells (FCs) are electrochemical reactors that convert hydrogen energy into electrical energy through a reaction that uses oxygen [1,2]. They are similar to flow batteries but can continuously produce electricity as long as the reaction is fed with fuel and oxygen. Power generation plants based on FCs represent one of the most promising and strategic technologies developed in recent decades because of their high energy conversion efficiency and environmental compatibility.

Proton exchange membrane FCs (PEMFCs) and solid oxide FCs (SOFCs) are the most widely used types of FCs [1,2], and they each have distinctive features, potential applications, and specific advantages and disadvantages. These two types of FCs are actually different electrochemical systems: PEMFCs are developed primarily for vehicular applications because they are lightweight systems that operate at approximately 80 °C, whereas SOFCs are preferred for distributed electricity generation because they operate at approximately 850 °C, providing high-quality waste heat to recover in cogeneration and bottoming cycles.

Unfortunately, FC-based power generation plants suffer from low reliability and limited lifetimes; thus, the development of specific methods for automatic on-line fault diagnosis is of paramount importance for their commercial diffusion and constitutes an active field of research [3,4]. The differences between PEMFCs and SOFCs, as well as the differences between the related power generation plants, result in significantly different malfunctioning mechanisms and fault typologies. Although numerous fault detection and isolation (FDI) methods have been developed for PEMFCs and related plants, as reviewed in [3], SOFCs and related plants have received insufficient attention until now [5,6,7,8,9]. The aforementioned differences make it impossible to directly apply the FDI methods developed for PEMFC plants to SOFC plants. 

In this study, we focus on SOFC-based power generation plants. Although these plants are not expected to perform load following, they should work both at the design point and in several off-design operating conditions, i.e., in numerous steady-state operating conditions. In addition, the faults that affect these plants are not binary events; rather, their size or extent can take on values in wide continuous intervals. Thus, to avoid efficiency losses and irreversible damages in SOFC plants, it is particularly important to rapidly detect and identify possible faults as soon as they occur irrespective of their size or extent and of the current operating condition.

Despite these needs, the vast majority of the FDI methods proposed for SOFC-based power generation plants [5,6,7,9] are designed to function under a single steady-state operating condition (or under a very restricted number of operating conditions) and for a small set of discrete sizes or extents of the considered faults. The reason for this limitation is that these FDI methods implement a conventional model-based FDI scheme in which the residuals (i.e., the differences between the measurements performed on the plant and the model predictions) are used for fault identification through an inference approach [10,11,12], namely, the fault signature matrix (FSM). This matrix associates each considered fault with a binary vector that has a number of elements equal to the number of residuals. If a given residual exceeds its threshold, then the corresponding vector element is equal to one; otherwise, it is equal to zero. As discussed in [13] for PEMFC plants and in [9] for SOFC plants, the use of binary codification of the residuals loses valuable information, and the strict association of each fault with a single vector does not allow all the possible combinations of fault sizes and operating conditions to be encompassed. As we will later show, if the SOFC plant is operated in many operating conditions and different fault sizes can occur (relevant assumptions in the application to real-world SOFC systems), then each fault can generate multiple binary vectors. Vice versa, there are specific binary vectors that can be obtained as the result of two or more faults. Therefore, the relationship between binary vectors and faults is many-to-many, which implies an intrinsic ambiguity, i.e., one cannot univocally determine the fault from the observation of the binary vector. Furthermore, from a pattern-recognition perspective, residuals are used as features for the detection and classification of faults, and thresholding a feature implies partitioning the feature space with a hyperplane orthogonal to one of its axes. Therefore, the decision regions corresponding to any decision rule that operates only on binarized features are constrained to be aligned with these hyperplanes. This is severely restrictive, especially as compared to a classifier that can operate with the original features and their joint statistics, and is allowed to determine arbitrarily shaped decision regions to maximize classification accuracy. Finally, the design of the threshold values is a critical task that significantly affects FDI performance [9]. 

Recently, an FDI method intended to overcome the aforementioned limitations in SOFC plants has been proposed in [8,14]. In this study, the residuals are processed through a statistical classifier that detects and identifies the faults in place of the thresholds and FSM. The introduction of a classifier and of a wide dataset to train such a classifier is typical of the data-driven FDI strategy (also called history- or knowledge-based strategy) [11,15,16,17,18]. This combination between the conventional formulations of model-based and data-driven FDI strategies constitutes another FDI approach, named hybrid or integrated strategy [16,18,19,20]. In [8,14], the statistical classifier is implemented through a support vector machine (SVM) [21,22], and the dataset is collected through an SOFC plant model in which the capability of simulating faulty conditions has been implanted. If we consider that the solution proposed in [8,14] represents a specific example from the large set of the potential combinations among pattern recognition techniques and FDI concepts (namely, data-driven and model-based schemes) in the field of SOFC plants, then it is evident that such combinations represent an open research domain. This study introduces new knowledge to the field of FDI systems for SOFC-based power generation plants by providing three original contributions:

(1) The definition of an FDI solution that is able to properly function in several operating conditions of an SOFC plant and for many sizes of each possible fault. To this end, model-based FDI schemes adopting the FSM, purely data-driven FDI schemes and hybrid FDI schemes, in which a supervised classifier is used to analyse residuals, are examined and compared.
(2) The assessment of the inclusion of physical quantities measured inside the SOFC stack among the variables used by the FDI system. Although the adoption of physical variables that are easy to measure in a real SOFC plant can yield satisfactory FDI performance, the impact of a few additional variables measured inside the SOFC stack is evaluated along with the current difficulties in measuring such variables.
(3) The introduction of random forests (RFs) [23], a powerful supervised nonparametric classification approach, as the pattern recognition technique to be used within a hybrid FDI approach in SOFC-based power generation plants. Although RFs have already been used in data-driven FDI for general machines [24,25], until now, they have not been considered in the FC field or in combination with a model-based FDI system. Here, we consider RFs because they offer some advantages that are particularly relevant in devising, developing, and testing FDI systems for FC-based plants.

The remainder of this paper is organized as follows: Section 2 describes the model-based, data-driven and hybrid strategies for fault diagnosis in SOFC plants and delineates the crucial role for FDI schemes of a plant model that is able to simulate both healthy and faulty conditions. Section 3 presents the SOFC plant structure and its operating conditions, the quantitative model, the considered faults, and the monitored variables in relation to the measurement difficulty. Section 4 introduces the supervised classification by RFs and the advantages with respect to other pattern recognition techniques. Section 5 reports and compares the results obtained using different FDI strategies and sets of monitored variables. Finally, Section 6 draws the conclusions of this study. 

## 2. FDI Strategies

### 2.1. Model-Based FDI

**Figure 1:**
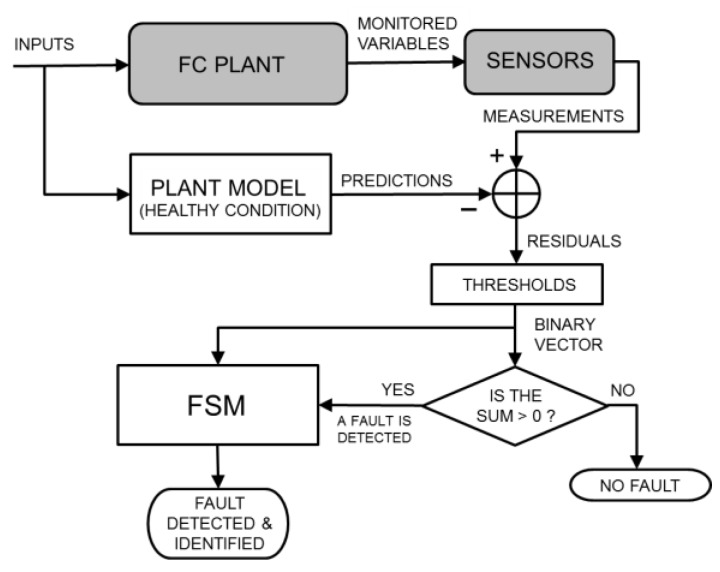
Schematic of the classical model-based FDI strategy for FC plants based on parity equations with output errors [12], residual binarization, and FSM.

Although the model-based FDI theory includes several schemes for the interaction between the real plant and the plant model [10,11], the parity equation scheme with output errors [12] is the preferred option for SOFC plants [5,6,7,9]. As shown in Figure 1, the plant and model receive the same inputs (which determine the plant operating conditions), and when no fault occurs, they are expected to produce the same outputs (which represent the monitored physical variables). In this condition, the residuals are zeros for less than the modelling and measurement errors. In the strategy commonly adopted for SOFC plants, the residuals are used to detect any possible fault through (absolute or relative) thresholds. Subsequently, the binarized residuals are used to identify the fault through an FSM, arranged based on a fault tree analysis, which is a deductive top-down tool and is typically used in safety and reliability engineering [6,7,9]. Because fault identification is performed using an inference approach, pattern recognition techniques are not involved in this task, and no dataset is necessary for training purposes. In this classical model-based FDI strategy for SOFC plants, the only possible use of pattern recognition techniques is to arrange a qualitative model for the plant, in case a quantitative model is not available or viable, as reviewed in [26,27].

### 2.2. Data-Driven FDI

**Figure 2:**
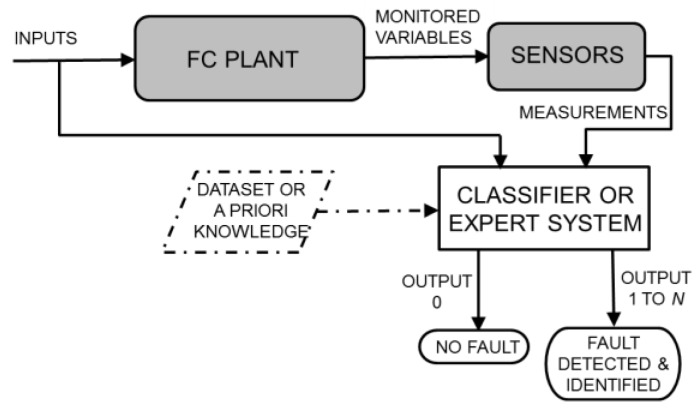
Schematic of the data-driven FDI strategy applied to FC plants.

To the best of the authors’ knowledge, a purely data-driven FDI strategy [11,15,16,18] has not yet been proposed for SOFC plants, although some data-driven schemes have been considered for PEMFCs and related plants, as reviewed in [28]. As shown in Figure 2, in the general data-driven scheme, both plant inputs and outputs feed the FDI system. The latter is implemented by pattern recognition or artificial intelligence techniques and should be adequately trained through statistically representative datasets or a priori knowledge. SVMs, neural networks and expert systems are the preferred tools for the fault diagnosis [18,29,30,31]. Unlike in the previous FDI strategy, a plant model is not required, and the fault detection and fault identification are potentially two undistinguished tasks (i.e., the plant healthy status represents a given class, such as each considered fault). In this study, we develop and test a data-driven FDI system by using the RFs to assemble a nonparametric supervised classifier. 

### 2.3. Hybrid FDI

**Figure 3:**
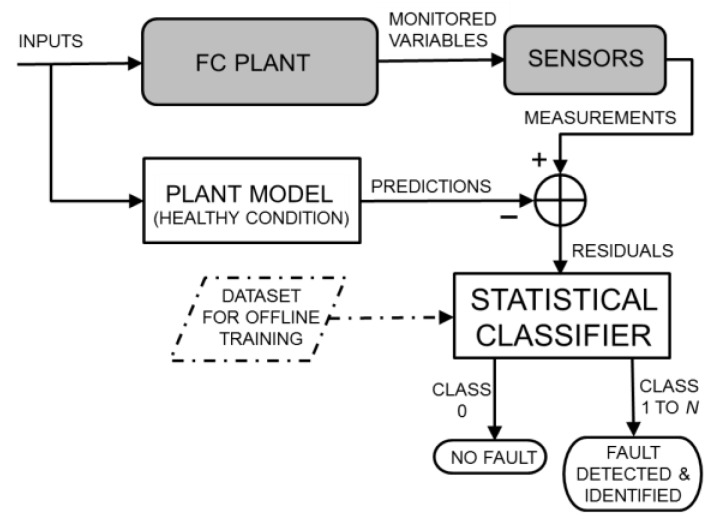
Schematic of the hybrid FDI strategy for FC plants, where residuals generated by the parity equations are processed by an adequately trained statistical classifier.

A possible combination of the two previous strategies leads to a hybrid FDI strategy in which, as illustrated in Figure 3, a plant model that receives the same inputs of the real SOFC plant is used to predict the monitored variables. The residuals generated by the parity equations with output errors [12] are used to detect and identify faults through a statistical classifier, such as that used in data-driven approaches, adequately trained offline using statistically representative datasets. Additionally, in this case, the fault detection and fault identification are two undistinguished tasks, preventing the need to set detection thresholds. Moreover, the classifier exploits the original values of the residuals without any binarization. In the FC field, this strategy has been preliminarily proposed in [8], where a quantitative model for an SOFC plant is used together with an SVM classifier and the FDI system is tested under ideal conditions (i.e., no errors). Subsequently, a joint model and feature selection technique has been proposed [22] to mitigate the computational burden that arises when modelling errors are introduced in the training and testing of the SVM classifier. 

In this study, we contribute to the progress of the hybrid FDI strategy for SOFC plants by proposing RFs as a powerful, flexible and time inexpensive technique for assembling the statistical classifier, thus enabling rapid training and testing of the FDI system in many different configurations. 

### 2.4. Plant Models Simulating Healthy and Faulty Conditions

**Figure 4:**
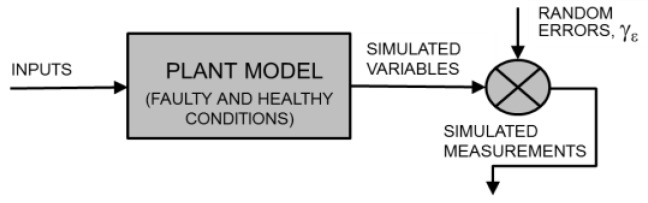
Replacement of the real FC plant and related sensors (see Figure 1, Figure 2 and Figure 3) by a plant model, which simulates healthy and faulty conditions and whose output variables are subject to random errors.

The requirement of a large amount of data for training the statistical classifier used in the data-driven and hybrid FDI strategies (i.e., a collection of the monitored variables or their residuals obtained under different healthy and faulty operating conditions and with various fault sizes) represents a practical problem. Not only is the collection of these data exceptionally time-consuming but the implanting of real faults in real systems also often produces irreparable damage with related economic loss. A similar problem occurs when the functioning of a conventional model-based FDI system should be assessed. Model-based FDI potentially offers a solution to this problem: if the model enables fictitious faults to be implanted inside it, such a model can be used to simulate a real plant operating under faulty conditions [8,9,22,32,33]. However, to also introduce model uncertainty and measurement tolerance, it is necessary to include random errors in the values of the physical variables simulated for the real plant. Each simulated variable can be multiplied by a random independent variable $\gamma_{\epsilon }$, uniformly distributed in [1−ϵ,1+ϵ], where 100 ϵ represents the maximum percentage error. In other words, the two grey blocks in Figure 1, Figure 2 and Figure 3 can be replaced by the blocks depicted in Figure 4, devoted to generating a realistic simulation of the monitored variables also in faulty conditions. The data obtained due to this replacement can be used to train the classifier (if any) and evaluate the performance of the FDI system. Therefore, the availability of a plant model that simulates healthy and faulty conditions is important not only for model-based but also for data-driven strategies, particularly during offline training and testing operations. 

The replacement of the real plant by a model is effective if the model is reliable and accurate. This requirement is typically satisfied when a quantitative mathematical model is deployed and has been validated with experimental trials that encompass several operating conditions. In addition, the inclusion of the physical equations that govern the real plant makes the quantitative model well suited for implanting fictitious faults with the required reliability and accuracy [4,34].

## 3. Measurements and Faults in the SOFC Plant and Related Model

### 3.1. SOFC Plant Structure and Operating Conditions

**Figure 5:**
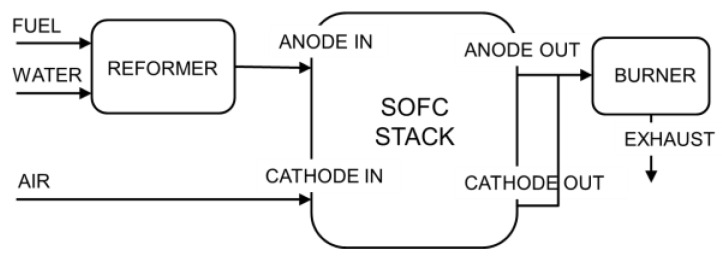
Schematic of the SOFC plant.

The laboratory-scale power generation plant that we consider in this study is composed of a reformer, an SOFC stack, and a post burner, as schematically illustrated in Figure 5. The reformer is fed with a mixture of methane and steam and converts the methane into hydrogen and carbon monoxide. These compounds are fed into the anode compartment of an SOFC stack while air is fed into the cathode compartment. The SOFC stack is composed of a number of rectangular planar cells superimposed onto each other. The electrochemical reaction produces steam and carbon dioxide along with electrical power and heat. The anode exhaust is mixed with the cathode exhaust and burned in the off-gas burner to reduce the release of pollutants and increase the temperature of the flue gas for further utilization.

The considered power generation plant is equipped with an inverter. In addition to its primary task of converting the DC electric power produced by the plant into AC electric power, the inverter controls the power supplied by the plant and the operational mode of the stack. The flow rates of the fuel, air and water entering the power generation plant are regulated by the controller to maintain the average temperature of the stack and the fuel utilization factor as close as possible to the desired values. These values determine which voltage-current characteristic curve for the SOFC stack is used. When the stack temperature and fuel utilization factor are maintained constant, different operating conditions can be defined moving along the characteristic curve. A potential fault affecting the SOFC stack is expected to distort such a curve, whereas faults affecting other plant components can modify the parameters that identify the characteristic curve. In both cases, the characteristic curve changes, and the inverter should control the plant in such a way to keep the generated current or the generated voltage constant. 

In this study, the inverter controls the plant to maintain the average temperature of the SOFC stack equal to 850 °C and the fuel utilization factor equal to 0.75. Under the design operating condition, the plant generates a voltage of 42.5 V and a current of 26 A (i.e., an electrical power of approximately 1.1 kW). Additional details regarding the structure and control of the considered SOFC generation plant are described in [4]. We focus on the constant-voltage control and define ten steady-state operating conditions (the design point and nine off-design points) by setting ten values for the generated voltage between 41 V and 50.5 V, and the corresponding current values range (for a healthy plant condition) between 30 A and 6.5 A, respectively. Overall, three inputs given to the plant: the desired voltage, average stack temperature and fuel utilization factor; however, only the desired voltage changes among the ten operating conditions. 

### 3.2. Monitored Variables and the Measurement Difficulties

The monitored variables that we consider for the fault diagnosis are the five physical quantities listed in Table 1. They are initially selected due to the ease with which they can be measured by a standard sensory suite deployed in the real plant. Although the internal parameters of the SOFC stack can potentially play an important role in understanding the plant health condition, none of the monitored variables come from the inside of the stack. The reason is the difficulty in developing sensors that can operate in the harsh conditions encountered inside the FC (in the case of SOFC, the high temperature) and without significantly affecting the chemical reactions that occur. Despite these difficulties, some recent papers propose and test sensor technologies for measuring the internal parameters of FCs during their operation. In particular, the measurement of the temperature and temperature gradient in SOFCs [35,36,37] has been proposed by adopting thin thermocouples, the measurement of temperature and humidity in PEMFCs [38,39] using multi-functional micro sensors, and the measurement of the losses due to the impedance increase inside the SOFC electrodes [40,41,42,43] using the electrochemical impedance spectroscopy. Therefore, in the final part of this study, we assume that the temperature gradient and the impendence losses can be measured inside SOFCs and introduce the maximum temperature gradient and the activation losses of the cathode as two additional monitored variables to assess the potential improvement in the performance of fault diagnosis system. 

### 3.3. Fault Classes

Concerning possible plant malfunctioning conditions, the following four fault classes are considered [4]: (1) SOFC stack degradation, i.e., a performance reduction due to the increase of the overall stack voltage loss due to internal problems, i.e., activation, ohmic and diffusion losses. It has been simulated by increasing the overall stack loss to between 105% and 160% of its nominal value; (2) Air leakage, i.e., a potential air leak between the air flow meter and the SOFC stack, reducing the flow that enters the stack to between 50% and 95% of the nominal rate; (3) Fuel leakage, i.e., a leak between the exit of the reformer and the entrance of the SOFC stack, which reduces the flow to between 30% and 95% of the nominal rate; (4) Reformer degradation, i.e., a lower conversion of methane due to catalyst degradation or carbon deposition, simulated by reducing the reaction to between 30% and 95% of the nominal rate. 

As shown in Figure 5, the SOFC plant under consideration includes an SOFC stack, a methane steam reformer, a burner, a fuel feeding system and an air feeding system. Because the burner is a well-tested and mature technology, its faults are not taken into account. The faults of the other four plant components are all considered, with one fault for each component. Because the different faults that can occur inside the SOFC stack provide similar effects [4], they have been combined into a single fault class. Similar considerations hold for the steam reforming reactor. Consequently, the FDI procedures discussed here cannot be used to distinguish among the different types of faults that occur inside the SOFC stack or in the methane steam reformer. If necessary, further investigations (for example, chemical or electrochemical tests) must be conducted to identify in detail the microscopic cause of the failure, as suggested in [4].

For each of the above faults, ten different sizes are considered by setting ten values that span the previously described ranges. Because each fault size is applied to each of the ten operating conditions, 100 combinations of operating conditions and fault sizes are identified for each fault. Overall, 400 combinations between the fault status (which includes the class and size of the fault) and operating conditions are considered in our investigation.

### 3.4. Quantitative Plant Model

The functioning of an SOFC-based power generation plant can be effectively simulated using a quantitative mathematical model, i.e., a model that is based on the physical equations ruling all the processes governing the plant, also called the “first-principle” or “white-box” model [26]. It can be obtained by coupling the models of the three main components (i.e., the FC stack, the reformer, and the burner). For the specific plant considered in this study, we adopted the model that has been extensively described in [4]. The reliability of the model predictions is assured by the model validation that has been performed with the experimental data collected during the operation of a real SOFC plant identical to the assumed one [4]. The plant was manufactured by Staxera GmbH (Dresden, Germany) [44] and was tested by EBZ GmbH (Dresden, Germany) within the project Genius [45], partially funded by the European Union. Five different steady-state operating conditions were considered, observing a maximum difference between the measured and predicted variables that was approximately 3% [4].

As previously mentioned, to preserve the experimental plant, the fault data can be obtained from a second version of the plant model that is modified to also simulate faulty conditions. To make the investigation more reliable, the model for the simulation of faulty conditions was also adequately validated. The validation exploited real data collected from the Staxera’s SOFC plant in which a limited number of faulty conditions were experimentally mimicked, as described in [34] and similar to [33].

Finally, although the quantitative models are generally considered to be computationally heavy, the described plant model is perfectly suited for working online in the configurations shown in Figure 1 and Figure 3. In fact, the FDI system that we consider is designed for steady-state operating conditions. This does not represent a limitation because transitions between operating conditions rarely occur in SOFC plants due to the slow transient response with characteristic times on the order of hours. SOFC systems are currently being studied primarily for applications as distributed power generation plants, where they are expected to work optimally at a given design point or otherwise in off-design operating conditions, which are still steady-state operating conditions. When the operating conditions change, it is only required that the plant model generate the prediction of the monitored variables before the real plant reaches the new steady-state condition. The proposed quantitative model satisfies this requirement. This model can potentially extend the functionality of the FDI to include transient conditions because the model is inherently dynamic [4], and its running times are significantly faster than those of the real system. For example, an experimental transient that is 2 × 10^4^ s in duration that occurs in various operating conditions is simulated in less than 10^3^ s on an Intel Xeon^®^ CPU (Intel Corporation, Santa Clara, CA, USA) operating at 2.93 GHz equipped with 6 GB of RAM [14]. 

## 4. Random Forests for Classification

### 4.1. RFs for FDI in SOFC Plants

The FDI task for a given SOFC-based plant is formalized as a supervised classification problem in which the set C of classes includes a finite number of distinct types of faults that can affect the plant and the non-faulty condition. The features used to discriminate these classes may be either measurements of physical or chemical variables taken on the SOFC (data-driven approach) or residuals between the measured and predicted values of these variables (hybrid approach). Let X be the resulting set of d features. As discussed in Section 2 and Section 3, the set ℒ of training samples to be used to learn a decision rule that discriminates among the classes in C is generated through the physicochemical model in [4]. The potentiality of the hybrid approach that combines supervised pattern recognition and physicochemical modelling has been demonstrated in [8,14] using a support vector classifier. Here, a different opportunity, based on the RF approach to classification, is investigated.

RF is well known as a topical and effective approach to supervised classification and regression [23]. It is rooted in the theory of multiple classifiers, which make use of ensembles of individual classifiers to exploit the complementarity among several (possibly numerous, simple, and weak) learning machines to determine powerful discriminant functions through decision fusion [46]. From a black-box perspective, SVM and RF, although they stem from different methodological approaches (kernel-based vs. ensemble learning), exhibit several common characteristics, including the fully non-parametric formulation and the applicability to data with arbitrary probability distributions. Their performances have often been compared [24,25,47,48] with each other and with those of alternate nonparametric supervised methods, such as artificial neural networks, with opposite conclusions on different specific applications but with overall comparable classification accuracies. Here, we propose RFs because, while they are expected to obtain accuracies similar to those of SVMs and other nonparametric techniques, they offer some advantages that are particularly relevant in operationally devising, developing and testing FDI systems for FC-based plants. These advantages consist of the small number of input parameters, the possibility of generally tuning these parameters in an efficient way, the generally short computation times, and the possible scalability and parallelizability.

### 4.2. RF Fundamentals

An RF is a suitable ensemble of tree classifiers, and a tree classifier is in turn defined by a set of decision rules sequentially interconnected by a tree topology [46,49]. Each node of each tree in the forest implies a decision rule that “splits” the classification process into two or more branches. The rationale is that although the rule associated with each node is often very simple (e.g., thresholding one of the features), the combination of many individually simple rules within each tree and the fusion of the outputs of an ensemble of trees within the forest allows a flexible and possibly complex overall decision criterion to be determined in the feature space [46].

Specifically, in an RF classifier, T trees are parameterized and trained in an independent and identically distributed (i.i.d.) manner. Denoting as θ a vector of parameters that characterize a generic tree classifier, any set of T trees whose parameter configurations $\theta_{1},\theta_{2},\ldots ,\theta_{T}$ are i.i.d. random vectors generally defines an RF [23]. Consistent with the well-known results of ensemble learning [46], the accuracy of the forest generally benefits from maximizing the diversity among the corresponding trees. To randomly generate the trees of an RF, a typical strategy, which has been found effective in numerous applications, is to use random sampling of both training data and features. For each tth tree, a set ${ℒ}_{t}$ of training data, including as many training samples as ℒ, is randomly drawn from ℒ uniformly and with replacement (bagging) and is used for training the tree itself (t=1,2,…,T). Denoting as $N_{t}$ the set of nodes of the tth tree, for each nth node ($n\in N_{t}$), a subset $X_{nt}\subset X$ of F features (1≤F≤d;t=1,2,…,T) is randomly drawn from X with a uniform distribution and is used in the decision rule of that node (random input selection) [23,46].

With regard to each individual tree classifier, the decomposition of the classification problem into the tree and the decision rule associated with each node may be defined, in principle, either heuristically, according to the user/operator’s prior knowledge about the addressed problem, or automatically on the basis of a training set. The latter approach is typically used in RF [49]. Here, binary trees are considered, the decision rule of each node consists of thresholding one of the features, and among the numerous supervised tree-based methods introduced in the literature (e.g., ID3, C4.5) [46], the CART (classification and regression tree) framework is used together with the aforementioned random sampling procedures. To construct the tth tree in the forest, CART is applied to the corresponding subset ${ℒ}_{t}$ of training samples to hierarchically determine the set $N_{t}$ of nodes, the feature $x_{nt}\in X_{nt}$ to be thresholded for each node $n\in N_{t}$, and the corresponding threshold value (t=1,2,…,T). This is accomplished by minimizing an index of the impurity, in terms of class membership, of the two regions of the feature space determined by the decision rule on the node. More details can be found in [46,49].

Given an unknown d-dimensional feature vector x to be classified, first, each tth tree is used to compute an estimate $y_{t}\in C$ of the class membership of x by applying the related sequence of decision rules to x while the tree is traversed from its root to its leaves (t=1,2,…,T). Then, majority voting is used to fuse the decisions expressed by the trees in the forest, i.e., $y_{t}$ is interpreted as a vote in favor of one of the classes, and x is assigned to the class with the most votes out of the T trees.

The number F of features to be drawn on each node and the number T of trees are the parameters of the method. The accuracy of RF is generally not critically sensitive to their values. T clearly impacts the execution time [23,46]. More details on the RF approach can be found in [23] along with the discussion of the generalization properties of the method.

## 5. Results and Discussion

In this section, we assess and compare the performance of the three previously described FDI strategies (see Figure 1, Figure 2 and Figure 3) when the same operating conditions and faults are considered. To perform an extensive test, the real plant is replaced by a quantitative model that is able to also simulate faulty conditions, subjected to random errors, as depicted in Figure 4. As mentioned above, the plant model was previously validated using data from a real SOFC plant under healthy and faulty conditions [4,34]. 

### 5.1. Simulated Measurements and Residuals

Because the prediction accuracy observed during the experimental validation of the SOFC plant model was approximately 3% and because the measurement tolerance is considerably smaller, we set the value for the maximum percentage error affecting each of the simulated variables (see Figure 4) equal to 4%. 

As described previously, 400 possible combinations are identified between the ten operating conditions and the ten sizes for each of the four faults considered. If independent, random errors are introduced for each simulated variable of each combination; then, a set of 400 vectors of simulated measurements (see Figure 4) can be generated. Because each fault is represented by 100 vectors, 100 additional vectors can be added for the healthy status. They can be obtained by repeating the random errors for each operating condition ten times. Thus, a complete dataset is composed of 500 vectors of simulated measurements. Successive independent random errors allow for the generation of an arbitrary number of these datasets.

For the FDI strategies that use a vector of residuals for the online diagnosis, such as those depicted in Figure 1 and Figure 3, the interaction between the model that replaces the real plant and the model that simulates the plant in healthy conditions enables the 500 vectors of simulated measurements to be transformed into just as many vectors of residuals. These residuals can be used to test the performance of a classical model-based FDI system, such as that depicted in Figure 1. However, the hybrid FDI system depicted in Figure 3 also needs an initial training phase. The residuals generated by some datasets can be used for this purpose, whereas the residuals generated by other datasets can be used for the performance test. 

The residual generation is not necessary for the data-driven FDI system depicted in Figure 2: some datasets of simulated measurements can be used for the training phase (assuming that a supervised classifier is adopted), and other datasets can be used for testing the diagnostic performance. 

### 5.2. Model-Based FDI with FSM

The FSM expresses the association between each fault and a binary vector whose elements represent the residuals after the thresholding procedure. This matrix is typically arranged using a fault tree analysis, which is a deductive top-down tool used at the component level and based on the knowledge of the components’ interactions at the system level [6,7,9]. Thus, in principle, the creation of the FSM does not require any collection of data or any plant model. However, when a statistically representative dataset is available, the FSM can be arranged from the analysis of the binarized residuals by associating to each fault the binary vector that is the most frequent when such a fault occurs. In this study, we will follow this option. 

**Figure 6:**
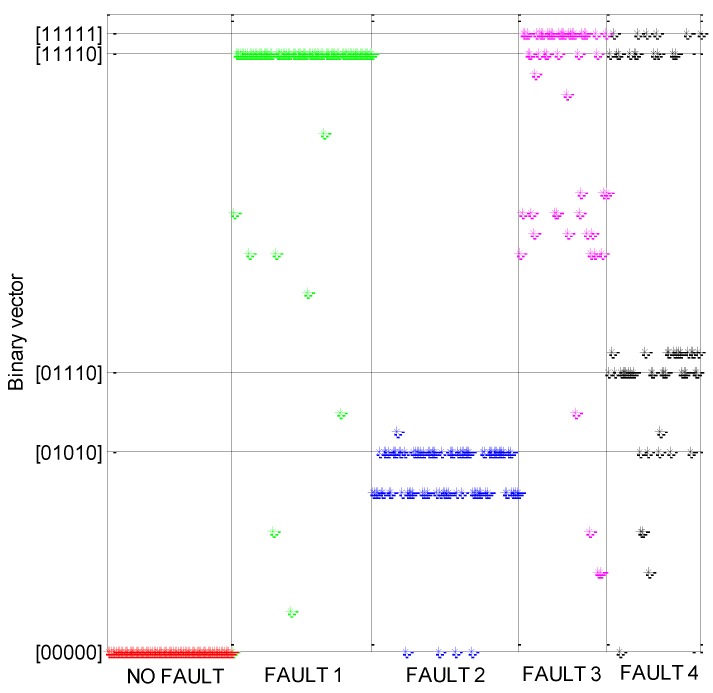
Binary vectors, from [0 0 0 0 0] to [1 1 1 1 1], produced by the model-based FDI system (see Figure 1) for the SOFC plant operating under healthy and faulty conditions. For a given fault, each asterisk indicates the binary vector produced by a given combination between the operating condition and fault size. The constant-voltage control for the SOFC plant is considered, the maximum percentage error is 4%, and the relative threshold Ω is 4.5%.

To investigate the binary vectors produced by the considered faults, a relative threshold [3,9] is set for each residual: the corresponding vector element is equal to one if the residual absolute value exceeds a percentage Ω of the predicted absolute value for that monitored variable; otherwise, it is equal to zero. Because the monitored variables listed in Table 1 are five, 32 different binary vectors can be defined, ranging from [0 0 0 0 0] to [1 1 1 1 1]. In our vectors, the most significant bit is related to the residual of the variable No. 1 in Table 1, and so on. Figure 6 shows the binary vectors obtained for the healthy status and for every faulty status when the 500 vectors of a dataset are used, and the relative threshold Ω is initially set to be equal to 4.5%. This figure shows that each fault produces more than one binary vector; two or three primary vectors can be identified for each fault. Additionally, the figure shows that different faults can yield the same binary vector. 

An accurate fault identification through the FSM is surely problematic in this situation, but this does not prevent achieving high performance in fault detection. If a fault is detected each time that the binary vector is different from [0 0 0 0 0] (see Figure 1), Figure 6 clearly shows that missed alarms (i.e., the fault is not detected when a fault is actually present) are very rare events. 

To maximize the fault identification performance, the FSM can be composed by associating each fault with the binary vector that has the maximum relative frequency for such a fault. Working in this way, the FSM presented in Table 2 is obtained. 

The definition of the FSM enables the assessment of the FDI system performance. All the datasets that have not been exploited to arrange the FSM can be used. Note that if a vector of residuals generates a binary vector that does not correspond to any of the five vectors inside the FSM, the identification of the related status (i.e., no fault, fault No. 1,…, fault No. 4) is not possible. Therefore, a fault is detected but is not classified. The relative threshold value is not necessarily equal to the value Ω used for arranging the FSM. Let us indicate with Ξ the percentage used as the relative threshold during the testing phase. Globally, the performance can be evaluated through the overall accuracy (OA), i.e., the fraction of test vectors whose status is correctly identified. We observed that the best OA is obtained when Ξ is between 4% and 5%. For Ξ belonging to this interval, an OA equal to 67% is obtained.

Clearly, the performance strictly depends on the FSM and in turn on the value Ω used to arrange such a matrix. If Ω ranges between 4% and 6%, then the association between faults and binary vectors is slightly different from that shown in Figure 6, but the maximum relative frequency occurs for the same binary vectors selected when Ω = 4.5%. Consequently, the FSM of Table 2 does not change and the OA is unaltered. 

In contrast, if the relative threshold Ω is set significantly higher than the maximum percentage error due to modelling and measurement inaccuracies (e.g., Ω = 9%), then the number of missed alarms dramatically increases and the problem due to the association of different faults with the same binary vector worsens. Conversely, if Ω is set slightly lower than the maximum percentage error (e.g., Ω = 3%), then a considerable number of false alarms (i.e., a fault is detected when a fault is not actually present) occurs and the number of binary vectors produced by each fault dramatically increases. In both cases, an FSM different from that of Table 2 is obtained, and irrespective of the value of Ξ, the OA is definitely lower than that obtained for the case of Ω = 4.5%, 4% ≤ Ξ ≤ 5%.

After many experiments are performed with different FSMs (i.e., different values for Ω) and using the value Ξ that maximizes the performance, we conclude that the best OA that is attainable for the datasets described in Section 5.1 does not exceed 67%. The adoption of the fault tree analysis to arrange the FSM has no actual possibility of improving this performance because we composed the FSM in such a way to maximize the OA for a given dataset. Although the latter is independent from the datasets used for the testing phase, it has the same statistical distribution of these datasets. Thus, the adopted FSM also represents the optimum choice for the test datasets. Instead, a performance improvement is potentially achievable by setting a specific threshold value for each residual. However, this optimization requires knowledge of the residuals’ statistics in both normal and faulty states, encompassing all the operating conditions, which is difficult to reach.

### 5.3. Data-Driven FDI with RF Classifier

In this study, we implemented the data-driven FDI strategy depicted in Figure 2 by deploying a statistical supervised classifier assembled through RF. This classifier operates by receiving the measurements of the monitored variables as input features. The training of the RF classifier can be performed using a dataset composed as described in Section 5.1. The remaining independent datasets are used, after normalization, for testing of the data-driven FDI system. 

The parameters used in the RF classifier are set as follows: T=10,F=3. Preliminary experiments, which are not reported for brevity, confirmed that these values allow for rather high accuracies to be obtained and that, most importantly, the accuracy of the classifier on the test set does not significantly change as T and F vary. For example, the accuracy remains remarkably stable as T varies from 10 to 100.

The obtained OA on the test set is equal to 72%. Although this value is 5% higher than that obtained with the FSM, this outcome cannot be considered a satisfactory result for the fault diagnosis task. Additionally, the performance for the basic fault detection task is poor: the relative frequency of correct fault detection is 91%, and the relative frequency of false alarms is approximately 27%. In particular, we note that the last value is unacceptably high. 

### 5.4. Hybrid FDI Strategy with Plant Model and RF Classifier

In the FDI strategy depicted in Figure 3, the statistical classifier operates by receiving vectors of residuals as input features. As in the data-driven case, the features should be preliminarily normalized to ensure that each feature has a zero mean and a unitary variance. The pool of independent (normalized) datasets is used as follows: one dataset is used for training of the RF classifier, and the remaining datasets are used for testing of the hybrid FDI system. 

The parameters used in the RF classifier are set as in the previous experiment with the data-driven approach (see Section 5.3). In this case, the obtained OA is equal to 86%. The relative frequency of correct fault detection is 96%, and the relative frequency of false alarms is approximately 7%. Therefore, valuable performance is obtained for both fault detection and fault identification. Actually, the higher accuracy obtained by this hybrid approach than by the model-based and data-driven approaches in Section 5.2 and Section 5.3 is an expected result because the former makes use of both supervised learning and physico-chemical modelling whereas the other approaches can only benefit from one of these two contributions.

Because this strategy provides the highest OA (19% higher than the traditional model-based OA and 14% higher than the data-driven OA), an additional investigation is performed. To evaluate the FDI performance for each specific status of the SOFC plant (i.e., no fault, fault No. 1,…, fault No. 4), the producer accuracy (PA) is introduced, i.e., the fraction of test vectors belonging to a given status that are correctly classified. Table 3 reports the PA of each considered status, showing that no fault, faults nos. 1 and 2 obtain a PA greater than or equal to the OA, whereas faults Nos. 3 and 4 obtain a PA lower than the OA. These faults are primarily confused with fault No. 1, which occurs with a relative frequency of 12% for fault No. 3 and a relative frequency of 19% for fault No. 4. This phenomenon is in accordance with Figure 6, where it can be observed that faults Nos. 3 and 4 occasionally produce the same binary vector associated with fault No. 1. 

### 5.5. Hybrid FDI Strategy Adding Variables Measured Inside the FC Stack

To achieve a potential improvement in the PA of the hybrid FDI system, the introduction of an additional monitored variable, which is measured inside the SOFC stack, is proposed. As hypothesized in Section 3, we assume that the temperature gradient inside the SOFC stack can be measured and introduce the maximum temperature gradient as a monitored variable in addition to the five variables listed in Table 1. Because the real plant is replaced by a quantitative model in our study (see Figure 4), this operation is easy. We repeated the generation of a pool of residual datasets (Section 5.1) and training of the RF classifier (Section 5.4) by considering six monitored variables in place of the previous five variables. The datasets not used for the training are used for testing of the FDI system. The achieved OA and PAs are summarized in Table 3: the OA value reaches 92%, showing a 6% increase with respect to the previous case, and the PA values are equal to or greater than those of the previous case. In particular, only for fault No. 3 is the PA lower than the OA, being equal to 85%. The inclusion of the maximum temperature gradient significantly improved both the OA and PAs, pushing their values above 92%, except the PA for fault No. 3 that was only marginally increased, from 82% to 85%. This problem holds because fault No. 3 is still confused with fault No. 1.

To investigate whether the introduction of another physical quantity measured inside the FC stack can improve performance, particularly the PA of fault No. 3, the measurement of the cathodic activation losses is assumed to be possible and is included as an additional variable. As in the previous case, the quantitative model used in place of the real plant makes it possible to implement this measurement, the generation of a pool of residual datasets, and the training of the RF classifier by considering seven monitored variables. The OA and PAs obtained by also using the cathodic activation losses are summarized in Table 3: the OA value is now 96%, and all the PA values are greater than or equal to 95%. The high performance reached in this configuration for the fault diagnosis task is associated with the likewise high performance for the fault detection task: the relative frequency of correct fault detection is 97%, and the relative frequency of false alarms is approximately 4%.

## 6. Conclusions

In this paper, we investigated different FDI strategies (namely, data-driven, model-based, and hybrid) for fault diagnosis in an SOFC power-generation plant that operates in numerous steady-state operating conditions and whose potential faults occur with random sizes. In this context, the impact of the potential inclusion of physical quantities measured inside the SOFC stack has been assessed. In addition, RFs have been investigated, for the first time, as the pattern recognition technique used in the FDI systems designed for SOFC plants. We concluded the following:

(1) Model-based FDI schemes that adopt the FSM and purely data-driven FDI schemes do not provide satisfactory performance when numerous operating conditions for the SOFC plant and many sizes for each fault are considered. Rather, a hybrid FDI strategy in which a supervised classifier is used to analyse residuals generated through a quantitative plant model can reach a high rate of fault detection and identification and a low rate of false alarms.
(2) The performance of the hybrid FDI strategy could be further improved by including, among the monitored variables, two physical quantities measured inside the SOFC stack, namely, the maximum temperature gradient and cathodic activation losses. Although the practical measurement of these quantities during the FC functioning is currently extremely difficult, recent research results provide the opportunity to achieve future solutions.
(3) RFs represent a suitable pattern recognition tool for achieving a supervised classifier that can be successfully applied for the FDI in SOFC-based power generation plants. In this context, the following advantages of RFs are particularly important: (i) RF is a fully non-parametric classifier, a property that makes it possible to jointly exploit the multisensor information associated with measurements of highly heterogeneous physical and chemical variables; (ii) in addition to the aforementioned high classification accuracy, RF has demonstrated remarkable computational efficiency with execution times of a few seconds to complete the training and testing tasks in all experiments; and (iii) the method includes only two parameters, which do not typically affect accuracy significantly and for which tuning is quite straightforward. All of these properties suggest that RF can be an effective choice as a supervised classification technique in the application considered in this paper. Based on our experience and several reports in the literature [24,25,47,48], we also note that alternate non-parametric classification techniques (e.g., SVM or artificial neural networks), while they can obviously obtain different accuracies with specific individual data sets on a case-by-case basis, are not expected to alter the validity of the general comments drawn in the previous points.

## Figures and Tables

**Table 1:** Monitored variables used for the FDI in the considered SOFC plant.

Variable	Physical Quantity
1	Generated electric power
2	Air flow rate entering the plant
3	Reformate fuel flow rate entering the SOFC stack
4	Air pressure loss between the inlet and outlet of the SOFC stack
5	Temperature at the burner outlet

**Table 2:** FSM of the model-based FDI system for the SOFC plant operating in constant-voltage conditions. The binary digits from *R*_1_ to *R*_5_ represent the residuals of the variables listed in Table 1 after the threshold operation.

Status	*R*_1_	*R*_2_	*R*_3_	*R*_4_	*R*_5_
No fault	0	0	0	0	0
Fault No. 1	1	1	1	1	0
Fault No. 2	0	1	0	1	0
Fault No. 3	1	1	1	1	1
Fault No. 4	0	1	1	1	0

**Table 3:** OA and PA obtained by the hybrid FDI system when the five monitored variables of Table 1 are used, when the maximum temperature gradient (MTG) inside the SOFC stack is added, and when the cathodic activation losses (CAL) inside the SOFC stack are additionally introduced.

Status	Variables 1 ÷ 5		Var. 1 ÷ 5, MTG		Var. 1 ÷ 5, MTG, CAL	
	PA		PA		PA	
No fault	93%	OA = 86%	93%	OA = 92%	96%	OA = 96%
Fault No. 1	87%		93%		95%	
Fault No. 2	94%		94%		96%	
Fault No. 3	82%		85%		99%	
Fault No. 4	75%		95%		96%	
